# Granular cell tumor of the breast during lactation: A case report and review of the literature

**DOI:** 10.3892/ol.2014.2563

**Published:** 2014-09-25

**Authors:** XIAJING QIAN, YIDING CHEN, FANG WAN

**Affiliations:** 1 Ningbo Medical Treatment Center, Lihuili Hospital, Ningbo, Zhejiang 315000, P.R. China; 2 Department of Surgery, Women’s Hospital, School of Medicine, Zhejiang University, Hangzhou, Zhejiang 310006, P.R. China

**Keywords:** granular cell tumor, breast, lactation, estrogen, prolactin

## Abstract

Granular cell tumor of the breast (GCTB) is a rare tumor, particularly in lactating women. This tumor can clinically and radiologically mimic breast carcinoma, which poses particular problems. The association between GCTB and sex hormones should receive particular attention. The present study reports a case of GCTB in a lactating patient. In this tsudy, the case of a 29-year old female who presented with a mass in the right breast is decribed. Immunohistochemical and cytological analysis revealed a GCT and subsequently wide local excision was performed. At 15 months following surgery, the patient is well and no tumor recurrence has been identified. A comprehensive review of the literature was also performed to assess and compare all cases of GCTB, with particular attention to hyperestrogenic and hyperprolactinemic states. Further studies are required to explore the association between granular cell tumors and hyperestrogenic and hyperprolactinemic states.

## Introduction

Granular cell tumors (GCTs), which were first described by Abrikossoff in 1926, are uncommon mesenchymal soft-tissue neoplasms (1,2). In 1931, Abrikossoff identified that, although they were originally noted in the tongue, GCTs were also associated with the breast (2). Female patients are more likely than their male counterparts to be affected. Patients of African descent have a higher incidence compared with those who are not of African descent (3). GCTs of the breast (GCTB) account for between 5 and 15% of all GCTs (4,5) and it is extremely rare to find the tumors in lactating females. GCTBs are difficult to distinguish from breast carcinoma by clinical, radiological or other observational techniques. Thus, pathological and immunohistochemical examinations are necessary (2). Although the association between GCT, estrogen and prolactin has not been proven, increased GCT incidence in the presence of hyperestrogenic and hyperprolactinemic states has been reported (3,6–10). The purpose of the present study was to review the management of GCTB and to present and discuss cases with GCT in the presence of hyperestrogenism or hyperprolactinemia. To the best of our knowledge, this report is the first Chinese case of a GCTB during lactation in the English literature. Written informed consent was obtained from the patient.

## Case report

**Figure 1:**
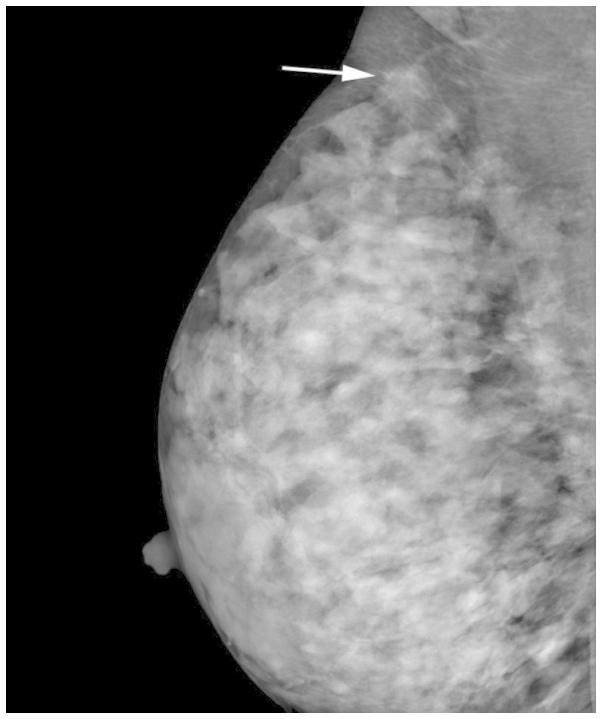
Mammography revealing an isodense and irregular spiculated mass (white arrow).

**Figure 2:**
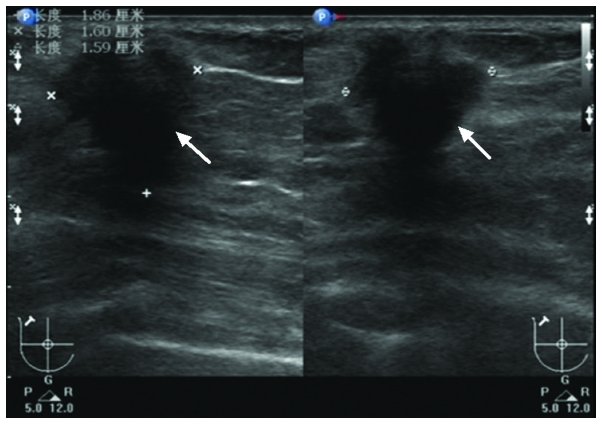
Ultrasound of the lesion showing a definite hypoechoic mass with angular margins, spiculations and an acoustic shadow posterior to the mass.

**Figure 3:**
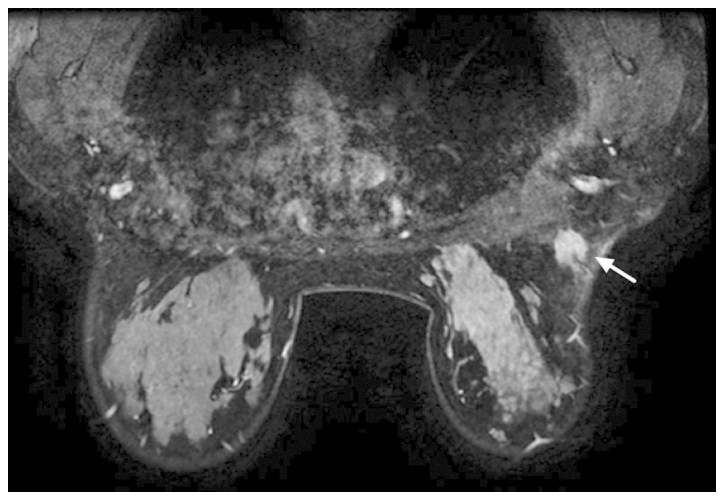
Dynamic magnetic resonance mammography of the mass revealing an irregular appearance.

A 29-year-old female presented to the Women’s Hospital (Hangzhou, Zhejiang, China) with a mass on the right breast, which had first been noticed four years earlier. The mass had increased in size in the latter half of the gestation and lactation periods. The patient had no medical history of malignancy. A physical examination of the breast revealed a firm, painless and vague nodularity in the upper-outer quadrant, near the axillary tail. Mammography revealed an isodense right-sided mass with ill-defined borders [Breast Imaging Reporting and Data System (BI-RADS) 4C; Fig. 1]. Ultrasound examination demonstrated a 1.9×1.6×1.6-cm hypoechoic, hypovascular and poorly-defined mass (BI-RADS 5; Fig. 2). Dynamic magnetic resonance (MR) mammography revealed homogeneous enhancement on a post-gadolinium-enhanced T1-weighted MR imaging (MRI) sequence and a high signal rim on a T2-weighted sequence (Fig. 3). The clinical and radiological findings were suggestive of malignancy.

**Figure 4:**
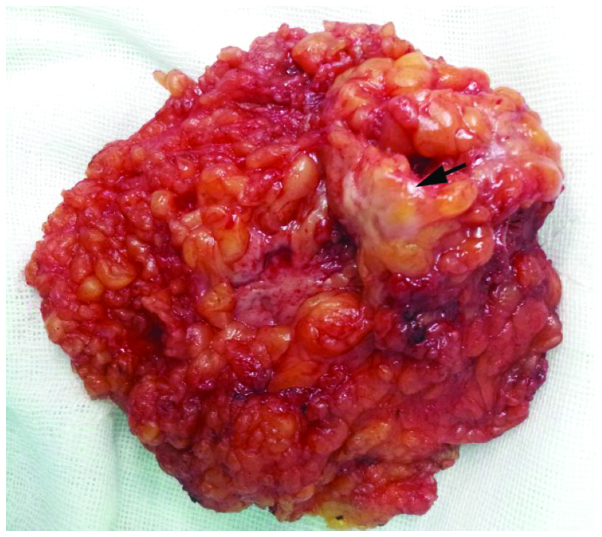
Cut surface of the resected specimen (black arrow) revealing a typical stellate-appearing fibrous area within fat tissue.

**Figure 5:**
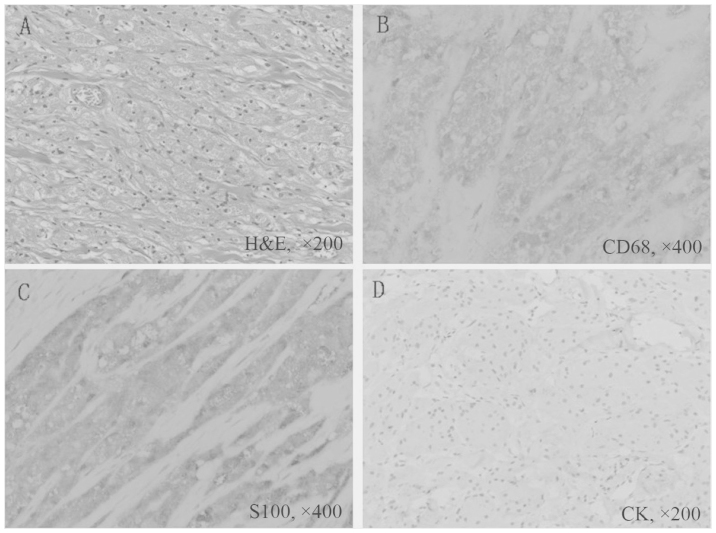
(A) H&E-stained GCTB sample (magnification, ×200). Mass cells with granular eosinophilic cytoplasm in groups within a dense collagenous stroma. (B) GCTB sample immunohistochemically stained for CD68 (magnification, ×400). CD68-positive mass cells. (C) GCTB sample immunhistochemically stained for S100 (magnification, ×400). S100-positive mass cells. (D) GCTB sample immunohistochemically stained for CK (magnification, ×200). CK-negative mass cells. GCTB, granular cell tumor of the breast; H&E, hematoxylin and eosin; CD, cluster of differentiation; CK, cytokeratin.

An ultrasound-guided core biopsy was performed and confirmed that the mass was a GCT, due to the cytological features observed and the immunohistochemical profile of the mass, thus, a wide local excision was performed. The intraoperative frozen section demonstrated that the mass was a GCT. On gross examination, the surgically excised specimen was comprised of fatty tissue fragments measuring 2.5×2.0×2.0 cm, and contained a firm, well-limited and yellow-white nodule (Fig. 4). Microscopically, the specimen was composed of nests or sheets of cells that contained cytoplasmic eosinophilic granules. The cells were generally uniform, large and polygonal. The nuclei were round to oval in shape, and the nucleoli were prominent (Fig. 5A). Immunohistochemical staining was strongly positive for cluster of differentiation (CD)68 (Fig. 5B) and S100 expression (Fig. 5C) and negative for cytokeratin expression (Fig. 5D). The GCT diagnosis was confirmed. The patient received no further treatment, and 15 months after surgery was in good health, with no tumor recurrence.

## Discussion

GCTBs account for between 5 and 15% of all GCTs (1,4,5). To the best of our knowledge, <400 cases of GCTBs have been reported in the literature. The tumors are most common in middle-aged premenopausal females and in African-American females (11), and occur largely in the upper-inner quadrant of the breast. However, in the present case, the mass was located in the upper-outer quadrant. GCTBs often present as a firm and painless mass, which may occur in the deep parenchyma, with fixation to the pectoral muscle, or in the subcutis, causing skin retraction, thus mimicking a malignancy (12).

Mammographically, GCTBs are known to exhibit a variable appearance, which ranges from well-circumscribed benign-appearing nodules to highly suspicious spiculated masses associated with skin retraction and thickening (13). Mammograms also often reveal GCTBs as stellate lesions without calcifications. The ultrasound appearance of a GCTB is usually a hypoechoic, ill-defined mass with posterior shadowing and a high boundary echo (14). A variety of MRI findings were illustrated in the cases reviewed in the present study, so no specific features of GCTB have been outlined. The patient in the present case exhibited all of the clinical and imaging features that have been classically associated with breast carcinoma, such as a firm and vague nodularity, an irregular spiculated mass on mammography and spiculated margins on sonography along with posterior shadowing. Irshad *et al* (13) reported spiculations as a common imaging feature that mimic carcinoma when present, and the present case was in agreement with this, since all the images exhibited a spiculated mass.

Although the clinical and radiological findings are mostly misleading, pathological investigations are essential in the diagnosis of GCTB. According to the European Society of Breast Cancer Specialists, pre-operative histological confirmation with a core biopsy can avoid mastectomy and axillary dissection (15).

GCTBs are usually firm and ill-defined masses with coloration ranging from white to tan (4,16,17). The majority of GCTBs are well-circumscribed lesions, but a significant proportion of them may be poorly circumscribed. Additionally, a lack of circumscription is now considered a common feature of GCTBs (18). Microscopically, the cytological features of GCTs include uniform cells, voluminous cytoplasm with fragile membranes, abundant granular eosinophilic cytoplasm and absent bare or bipolar nuclei (2). GCTs are also generally uniform, large, bland and polygonal, and are arranged in nests and sheets. Granular change is caused by the cytoplasmic accumulation of lysosomes. GCTs do not exhibit mitoses, pleomorphism, nuclear multiplicity or atypia (19,20). Immunohistochemical staining is useful in differentiating GCTBs from mammary carcinoma. The tumor cells in GCTs are strongly immunoreactive to S100 and CD68. CD68 expression is an immunohistochemically distinctive feature of GCTs and is associated with an abundance of phagolysosomes (20). S100 is a sensitive marker for GCTB, but it is not specific, as certain breast malignancies are also S100-positive (21). CD68 and S100 stain negative for cytokeratins, epithelial membrane antigen and mucin (22). CD68 and S100 aid in the differentiation between a GCT and apocrine carcinoma.

The factors affecting the development of GCTB have not been clearly confirmed, and the association between GCTB and hormones remains inconclusive. Certain cases have reported that GCTs occur during pregnancy and in hyperestrogenic and hyperprolactinemic states (3,4,7–10,23,24). In the present study, the size of the tumor increased rapidly in the latter half of the gestation and lactation periods, and the levels of estrogen and prolactin were high. Review of the literature revealed five other cases of GCTs in which a history of increased bodily estrogen was noted at the time of diagnosis. Kommoss *et al* (4) reported a GCTB in a 20-year-old, pregnant female of African descent. Ipakchi *et al* (3) noted a patient with recurrent GCT in subsequent pregnancies during the later stages of pregnancy. Yang *et al* (23) reported the case of a 5-month pregnant, 21-year-old patient who was found to have a malignant mediastinal GCT. Mahoney *et al* (7) reported a thyroid GCT in a nine-year-old patient receiving high-dose estrogen therapy. Benisch *et al* (8) reported the case of a GCT of the trachea in a first-trimester, 25-year-old patient. The review of the literature revealed three additional cases of GCT in which a history of increased prolactin was noted at the time of diagnosis. Lee *et al* (9) reported a GCT of the neurohypophysis in a 36-year-old female whose laboratory examination revealed hyperprolactinemia. Higuchi *et al* (10) noted a hypophyseal GCT case, which presented as visual failure and hyperprolactinaemia (serum prolactin level, 274 ng/ml; normal, <10 ng/ml). Popovic *et al* (24) reported a case of GCT of the sellar region with hyperprolactinemia. However, GCT cells are also negative for estrogen and progesterone. The hormonal effects on GCT have not been completely investigated.

Wide local excision is the recognized treatment for GCTB. The local excision of lymph nodes or a sentinel lymph node biopsy is not indicated, except in the case of malignant GCT (1).

GCTB is a rare tumor that mimics breast malignancy clinically and radiologically. Pathological correlation usually clarifies the diagnosis, but further examination via immunohistochemical staining is necessary. Although an association between GCT, hyperestrogenism and hyperprolactinemia has been postulated, the small number of studies investigating the hypothesis precludes any definitive association.
